# A Case Report of HIV‐Associated Pituitary Lymphoma and Review of the Literature

**DOI:** 10.1002/ccr3.71546

**Published:** 2025-12-02

**Authors:** Yahui Cui, Yuxiao Yuan, Liangyan Jin, Xiantu Zhang, Hui Hou

**Affiliations:** ^1^ Department of Neurosurgery Hangzhou Xixi Hospital Hangzhou China; ^2^ Department of Medical Imaging Hangzhou Xixi Hospital Hangzhou China; ^3^ Department of Clinical Pharmacology Hangzhou Xixi Hospital Hangzhou China; ^4^ Department of Pathology Hangzhou Xixi Hospital Hangzhou China

**Keywords:** HIV, hypopituitarism, lymphoma, pituitary gland

## Abstract

Primary saddle region tumors are the most commonly pituitary adenomas: They account for more than 90% of all saddle region tumors. Primary central nervous system lymphoma (PCNSL) lesions are relatively rare and are usually found around the ventricles. Reports of PCNSL lesions located in the saddle region are even rarer. We report a rare case of PCNSL originating from the pituitary–pituitary stalk–mammillary body region in a 33‐year‐old man. Clinical manifestations included progressive endocrine abnormalities and cavernous sinus syndrome. His initial symptoms consisted of fatigue, nausea, vomiting, and diplopia. Endocrine investigations revealed anterior pituitary hypopituitarism, with secondary hypothyroidism, hypogonadism, and adrenal insufficiency. Magnetic resonance imaging (MRI) showed an enhancing lesion in the pituitary stalk and pituitary gland involving the right cavernous sinus, along with loss of the posterior pituitary's T1 hyperintensity. The initial diagnosis at the external hospital was nonbacterial inflammatory pituitary disease. After treatment with methylprednisolone, some symptoms improved, and MRI scans indicated a slight reduction in lesion size. However, the diplopia did not resolve. Two weeks after steroid withdrawal, the patient's symptoms worsened. Upon referral to our hospital, he tested positive for HIV, and follow‐up MRI demonstrated lesion enlargement with necrosis and hemorrhagic infarction, as well as reappearance of the posterior pituitary T1 hyperintensity. Following hormone replacement therapy, endoscopic transsphenoidal surgery was performed, and histopathological examination confirmed B‐cell lymphoma.

## Key Clinical Message

Acute hypopituitarism with abnormal enhancing lesions of the hypothalamus–pituitary stalks on imaging requires consideration of the possibility of pituitary lymphoma. A definitive diagnosis requires histopathology.

## 1 Introduction

Central nervous system (CNS) lymphoma is a rare disease, accounting for about 3%–4% of newly diagnosed CNS tumors [1]. It occurs in a variety of anatomical regions, including the basal ganglia, thalamus, corpus callosum and periventricular white matter. However, hypothalamus involvement is rare [2]. Primary pituitary lymphoma is extremely rare. Of 1120 patients with transsphenoidal surgery for saddle region masses, only one (0.1%) was reported to have primary pituitary lymphoma [3]. In another report, of 353 patients with a presumptive diagnosis of pituitary tumor, only one (0.3%) was confirmed to have a primary pituitary lymphoma [4].

Most PCNSL tumors belong to a highly aggressive diffuse large‐cell subtype, usually with a B‐cell phenotype of origin [5, 6, 7]. The clinical presentation of CNS lymphomas varies depending on the location and size of the tumor. Endocrine abnormalities are common in hypothalamus–pituitary (HP) lymphomas, and the most common clinical manifestation is hypopituitarism. An important risk factor for the development of PCNSL is immunodeficiency, and PCNSL in the HIV population has been observed mainly in the context of advanced disease. More common when CD4 counts are less than 50/μL and have atypical lesions and rapid progression on imaging [8, 9]. In this article, we present a case with the first presentation of anterior pituitary hypopituitarism with cavernous sinus syndrome, in which HIV was detected on preoperative screening and histopathology after pituitary occupancy resection suggested a rare pituitary lymphoma.

## 2 Case History/Examination

**FIGURE 1:**
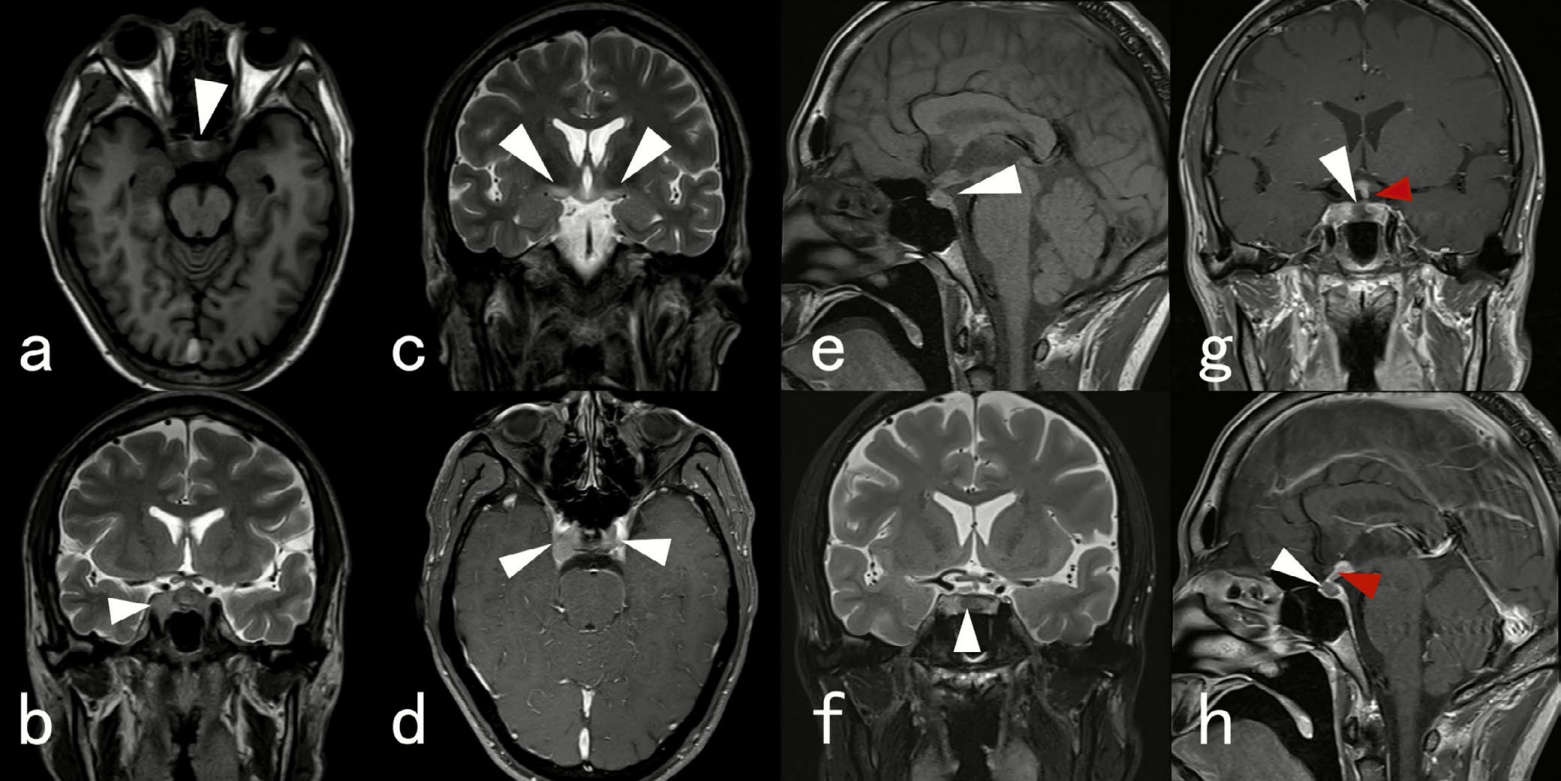
First hospital MRI: (a) high signal intensity on T1‐weighted imaging (T1WI) as indicated by the arrow. (b) Mass‐like T2‐weighted signal (T2WI) lesions visible paranasophally and suprasellar, with bilateral cavernous sinus involvement. (c) Symmetrical edema visible in both thalami. (d) Marked enhancement observed on contrast‐enhanced imaging. Second hospital MRI. (e) Indicates pituitary gland fullness with prominent posterior lobe, upward bulging, height approximately 7.6 mm. T1WI shows no marked hyperintensity in posterior lobe. (e, f) Pituitary signal appears slightly hypointense on T1WI and T2WI. (g, h) White arrows indicate no significant enhancement on contrast‐enhanced scan. Red arrows indicate uneven thickening of the pituitary stalk with localized leftward displacement; the stalk demonstrates markedly heterogeneous enhancement on contrast‐enhanced imaging.

A 33‐year‐old male patient presented with nausea, vomiting, malaise, blurred vision accompanied by diplopia, and ptosis of the right upper eyelid for more than 1 month prior to admission. The patient had no previous history of chronic disease or specific medications. He is a teacher by profession and is unmarried. Upon initial admission to the first hospital, an enhanced MRI of the skull suggested parasellar and suprasellar masses, with a possible pituitary tumor and Langhans cell hyperplasia pending further evaluation. He was subsequently referred to a second hospital with a diagnosis of pituitary gland inflammation. Laboratory results indicated a thyroid‐stimulating hormone (TSH) level of 0.01 nmol/L, total triiodothyronine (TT3) of 0.89 nmol/L, and free thyroxine (FT4) of 7.46 nmol/L. The patient received methylprednisolone therapy and thyroxine supplementation. A review of the pituitary enhancement MRI revealed a full pituitary gland, with a markedly prominent posterior lobe measuring approximately 7.6 mm in height. The T1‐weighted imaging of the posterior lobe did not show significant high signal intensity, while the pituitary signal was slightly low on T1‐weighted imaging and T2‐weighted imaging; no obvious enhancement was observed on the enhancement scan. There was uneven thickening of the pituitary stalk with localized left deviation and marked uneven enhancement on the enhanced scan (Figure 1). The patient was discharged with improved clinical symptoms. However, the aforementioned symptoms worsened again 1 week ago and he was referred to our hospital. Physical examination, the patient's temperature was 36.6°C, heart rate was 79 beats per minute, and blood pressure was 105/77 mmHg. There was mild ptosis of the right upper eyelid, an inability to abduct the right eye, and no obvious defects noted in the gross measurement of the visual field. The patient was conscious and alert, exhibiting a soft demeanor, and muscle strength in the limbs was normal.

**FIGURE 2:**
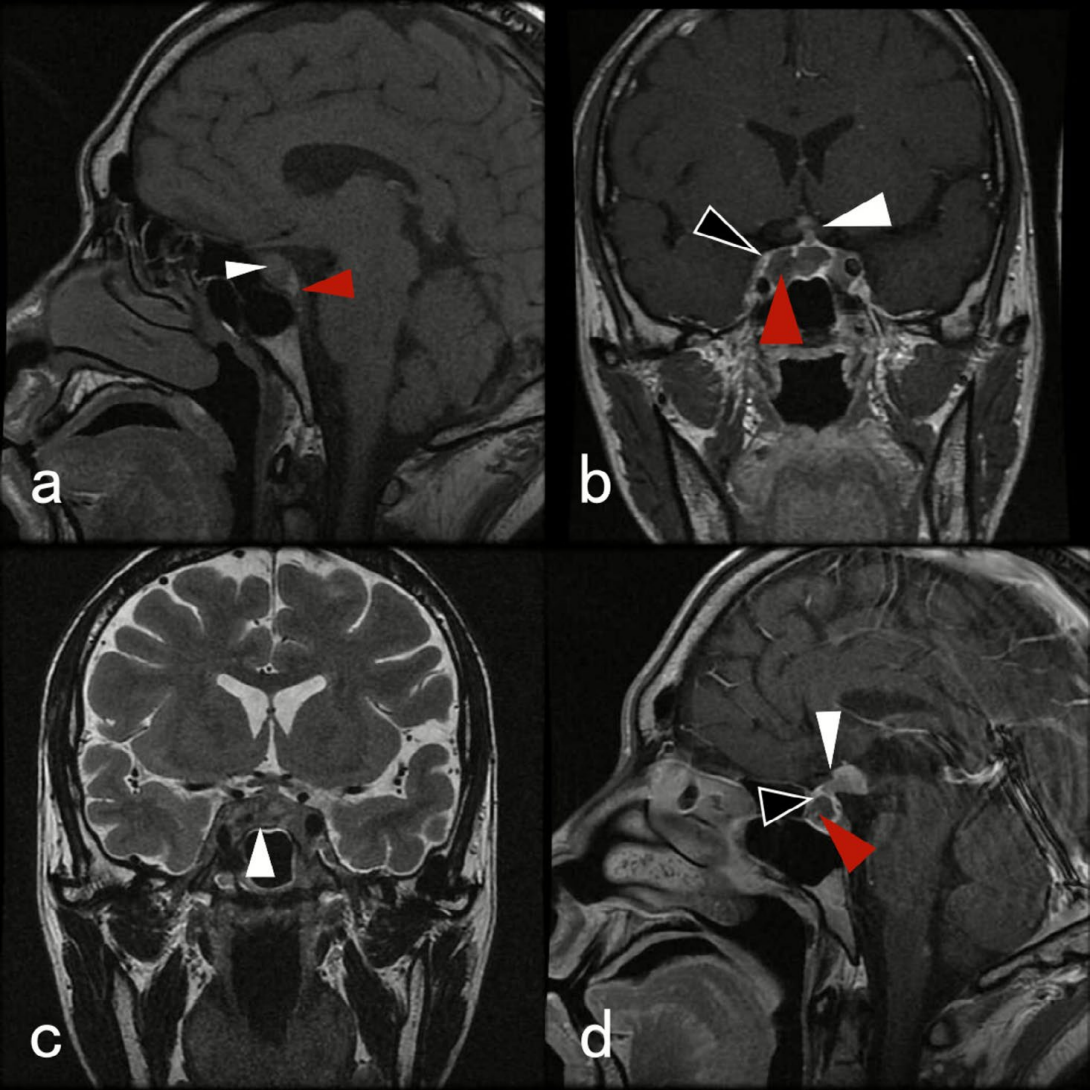
Preoperative magnetic resonance examination (a, b, c, d). Pituitary gland significantly enlarged in coronal view, measuring approximately 13 mm in height. (a) Red arrow indicates hyperintense signal in posterior pituitary lobe. (a, b) White arrows indicate predominantly slightly hyperintense signal on T1‐weighted images (T1WI) and mixed hyper‐ and hypointense signal on T2‐weighted images (T2WI). No sella turcica depression is present. The pituitary partially encircles the internal carotid arteries laterally and extends superiorly to near the level of the optic chiasm. The pituitary stalk is shortened and thickened with a nodular appearance. Above the stalk, nodular lesions with isointense signal on both T1 and T2 sequences are noted at the level of the gray nodule and the mastoid process, measuring approximately 13 × 9 mm; (b, d) hollow arrows indicate marked enhancement of the lesion margins within the saddle region on the contrast‐enhanced scan; red arrows denote the short T1 signal with no enhancement within it (dimensions approximately 11 × 22 × 13 mm). White arrows indicate mild enhancement of the pituitary stalk, with marked persistent enhancement of the gray nodule and the nodule at the mastoid process.

Table 1 suggests that the patient presented with hypokalemia and anterior pituitary hypopituitarism. To address hypothyroidism, a hydrocortisone intravenous infusion of 100 mg was administered every 12 h alongside thyroxine tablets. The pituitary enhancement MRI indicated significant enlargement of the pituitary gland, measuring approximately 13 mm in height. The coronal and sagittal views revealed a slightly high signal in most of the T1‐weighted images (T1WI), and mixed high and low signals in the T2‐weighted images (T2WI). The pituitary stalk appeared shortened and thickened in a nodular configuration, with nodular isotropic T1WI and isotropic T2WI observed in the gray nodule and papillary body above the pituitary stalk, measuring approximately 13 × 9 mm. Enhancement scans demonstrated significant enhancement at the edges of the lesion in the sella, while no enhancement was noted in the short T1WI signal within the sella which measured approximately 11 × 22 × 13 mm. Mild enhancement of the pituitary stalk was observed, and the nodules in the gray nodule and papillary body exhibited significant and persistent enhancement. A pituitary tumor with stroke is the primary consideration, necessitating identification of gray nodules and papillary hyperplasia (Figure 2). Following pharmacological treatment, cortisol levels returned to normal. A comprehensive lymph node ultrasound revealed only slight enlargement of lymph nodes in both sides of the neck, with no significant abnormalities observed in the liver, spleen, or other organs.

**TABLE 1:** Laboratory values observed at initial consultation.

Component	Result	Reference range
Leukocyte	9.62 × 10^9^/L	3.5–9.5 × 10^9^/L
Lymphocyte	3.46 × 10^9^/L	1.1–3.2 × 10^9^/L
Potassium (K)	3.02	3.5–5.3 mmol/L
Sodium (Na)	146.2	137.0–147.0 mmol/L
Prolactin	5.65	3.42–19.15 ng/mL
Growth hormone (GH)	0.55	0.06–5 μg/L
Thyroid‐stimulating hormone (TSH)	< 0.01	0.56–5.91 μIU/mL
Free triiodothyronine (fT3)	0.28	0.08–0.19 ng/dL
Free thyroxine (fT4)	0.77	0.61–1.64 ng/dL
Follide‐stimulating hormone (FSH)	0.06	0.95–11.95 mIU/mL
Estradiol (E2)	< 10	11.0–44 pg/mL
Luteinizing hormone (LH)	0.04	0.57–12.07 mIU/mL
Testosterone (TE)	< 0.45	4.94–32.01 nmol/L
Adrenocorticotropic hormone (ACTH) 8 am	7.1	7–65 ng/L
Cortisol 8 am	27.74	176.58–629.05 nmol/L
Adrenocorticotropic hormone (ACTH) 16 pm	2.4	3.40–35.4 ng/L
Cortisol 16 pm	143.29	< 275.9 nmol/L
Adrenocorticotropic hormone (ACTH) 0 am	4.44	Am 7–65 ng/L, pm 3.40–35.4 ng/L
Cortisol 0 am	50.92	am 176.58–629.05 nmol/L, pm < 275.9 nmol/L
Immunoglobulin G4 (IgG 4)	205	36–2090 μg/mL
HIV RNA	1.18 × 10^6^	< 58 copies/mL
Urinary density	1.004	1.003–1.030
CD4 count	406	432–1341 cells/μL

## 3 Outcome and Follow‐Up

**FIGURE 3:**
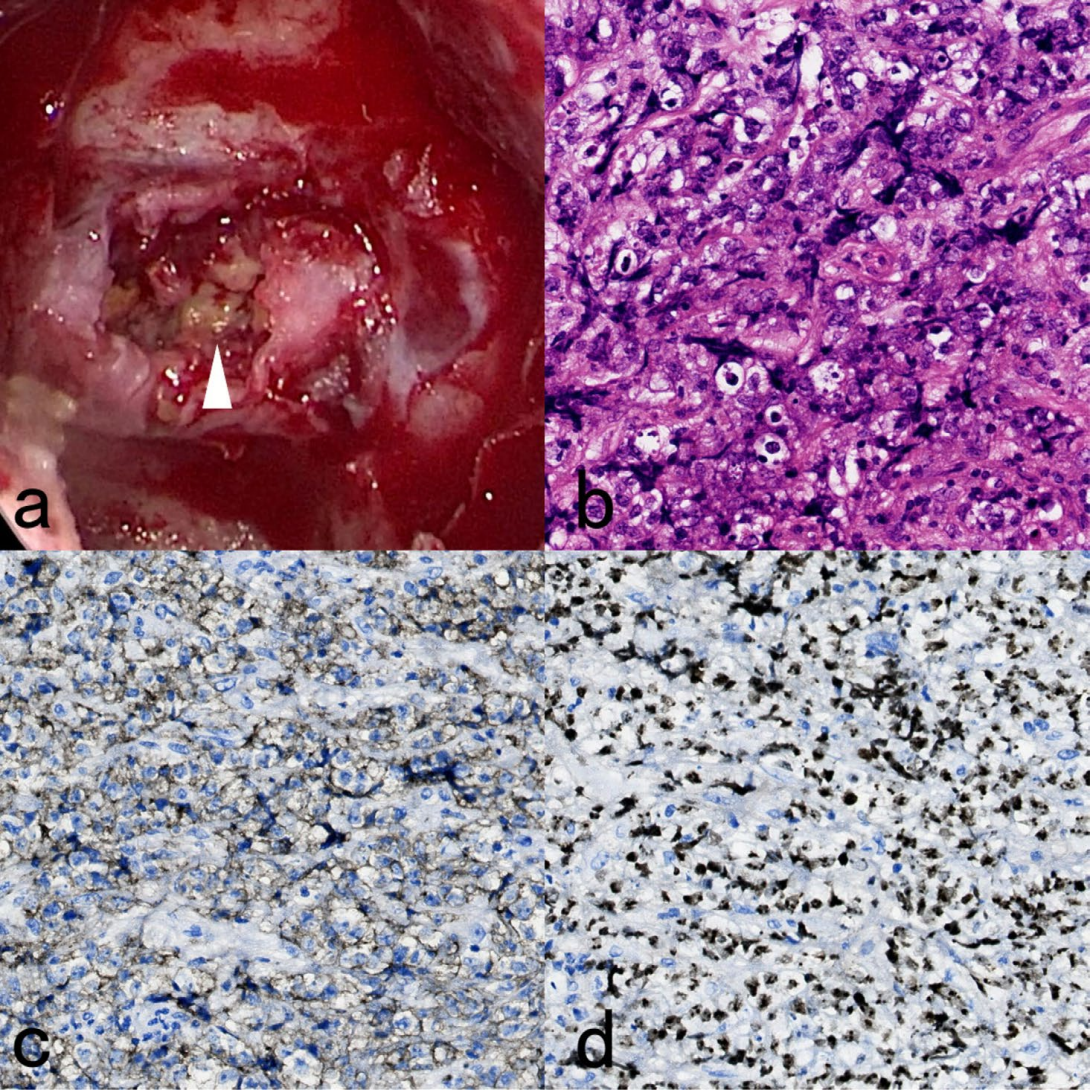
(a) The white arrow shows the yellowish‐white tumor tissue seen intraoperatively, which is hard and tough. (b) High‐powered field, hematoxylin, and eosin stain; 40×. (c) Immunohistochemistry‐positive neoplastic lymphoid cells for CD20; 40×. (d) Ki67 immunohistochemistry indicated a high proliferative index of tumor cells, approaching 90%; 40×.

A multidisciplinary discussion involving neurosurgery, endocrinology, and radiology concluded that a resection and biopsy of the pituitary lesion were warranted. Intraoperatively, the tumor tissue was characterized as yellowish‐white, hard and tough. Postoperative pathology revealed extensive necrotic tissue and residual adenohypophyseal shadows in the sella turcica, accompanied by peripheral inflammatory tissue hyperplasia with local aggregates of medium‐sized heterogeneous B lymphocytes. Immunohistochemical analysis showed positivity for CD20+ and CD34+, a Ki‐67 proliferation index of 90%, negativity for P53, and scattered positivity for synaptophysin. Considering the patient's history of HIV and corticosteroid use, a diagnosis of B‐cell‐derived lymphoma or B‐cell lymphoblastic lymphoma was proposed (Figure 3).

Postsurgery, the patient received intravenous hydrocortisone infusion and oral thyroxine tablets to address endocrine dysfunction, during which transient central diabetes insipidus occurred. Administration of posterior pituitary hormones was gradually tapered over 72 h. Concurrently administer Bictegravir sodium, emtricitabine, and tenofovir alafenamide fumarate tablets (HAART therapy). The patient was recommended to pursue further investigations, including molecular pathology tests, to clarify the specific pathology. However, the patient declined any additional investigations or medications and left the hospital without follow‐up.

## 4 Discussion

Tumors in the saddle region are very common, usually benign, and 90% are pituitary adenomas [10]. The next contains craniopharyngiomas, germ cell tumors, meningiomas, and chordomas. Most pituitary tumors are asymptomatic, but depending on the cell type, they can be due to the behavior of the tumor cells in terms of hormone secretion and growth. They may show different symptoms. Nonfunctioning pituitary adenomas usually produce symptoms secondary to the occupying effect, which may include hypopituitarism due to compression of pituitary structures, in addition to other symptoms such as headaches and visual disturbances. PCNSL is a subtype of non‐Hodgkin's lymphoma which is rare in immunocompetent patients. However, the incidence has increased in recent years. PCNSL accounts for 1% of all lymphomas, 4%–6% of all extranodal lymphomas, and approximately 3% of all CNS tumors [11]. PCNSL is usually found in the periventricular and basal ganglia. Lymphoma involvement confined to the saddle or parasaddle region is a rare disease. In a literature review reviewing the characteristics of 32 saddle zone lymphomas, only 2 of 32 cases involved both the hypothalamus and pituitary stalks, and 19 of 30 (63.3%) had lesions confined to the saddle zone. None of the cases involved both hypothalamus–pituitary stalk–pituitary. It was also found that 25 of 30 (83.5%) of the lymphomas in this region had pituitary dysfunction, except for two cases that were not described. Moreover, hypothalamic lesions were found to be associated with both anterior and posterior pituitary dysfunction, whereas anterior pituitary dysfunction was predominantly associated with anterior pituitary dysfunction in saddle region lesions [12]. Our case did not present with posterior pituitary lobe dysfunction, although there were early posterior pituitary lobe signaling changes and a hypothalamic–pituitary stalk–pituitary lesion.

The sella turcica, owing to its anatomical location, lies in close proximity to the cavernous sinus, optic nerve, and cranial nerves near the clivus. Of these, the optic and abducens nerves are most likely to be involved. When the lesion is located in the saddle region, the second and sixth cranial nerves can cause dysfunction. Lesions located in the hypothalamus and pituitary stalks tend to cause dysfunction of the second cranial nerve [12]. PCNSL with concomitant hypothalamic–pituitary stalk–pituitary lesions is extremely rare [13]. In this case, the lesion was located in the hypothalamus–pituitary stalk–pituitary gland, but showed dysfunction of the third and sixth cranial nerves due to the invasion of the right cavernous sinus by its main body.

The clinical presentation of pituitary lymphoma lacks specificity, especially in immunodeficient patients with atypical imaging, and is often confused with pituitary adenoma, pituitary inflammation or germ cell tumor [14]. It is noteworthy that this patient presented with anterior pituitary hypopituitarism and cavernous sinus syndrome at the beginning of the disease. However, the occupancy was not significant on imaging, with signal abnormalities in the hypothalamus, pituitary stalk, and pituitary gland, and signal changes in the posterior pituitary lobe on MRI T1WI. The clinical diagnosis was pituitary inflammation [15, 16]. It may be related to the direct invasion of the right cavernous sinus by the tumor and affect the normal secretion of the pituitary gland. Although the imaging suggests that the hypothalamus papillary body and pituitary stalk have abnormal reinforcement signals, it does not show the abnormal function of the posterior pituitary gland. After the patient was treated with methylprednisolone, the posterior pituitary lobe recovered high signal on MRI T1WI, which may suggest that it is little infiltrated by tumor cells. On imaging, pituitary lymphoma tends to be equal or low signal on MRI T1WI and equal or slightly high signal on MRI T2WI, and enhancement scans often show uniform enhancement, which is different from the cystic or hemorrhagic tendency of pituitary adenomas. However, in this case, the patient was treated with hydrocortisone and developed partial necrosis with hemorrhagic manifestations, which made the differential diagnosis even more difficult to rely on imaging alone, and ultimately the diagnosis had to be confirmed by histopathology and immunohistochemistry (e.g., positive B‐cell markers such as CD20) [17]. With the rapid development of artificial intelligence, the future is expected to change the diagnostic paradigm through deep computer learning [18].

HIV infection leads to significantly higher incidence of lymphoma [19]. Almost all cases of PCNSL are diffuse large B‐cell lymphomas, although the pathogenesis is unclear. Like PCNSL, primary pituitary lymphoma is most commonly of B‐cell origin. People living with HIV in particular, are susceptible to activating attacks such as EBV and continuous chronic antigenic stimulation due to increased CD4+ T‐cell destruction, which indirectly affects the regulation of B‐cell proliferation. Multifactorial influences such as cytokine secretion and other factors leading to B‐cell overactivation and inhibition of apoptosis result in a marked increase in B‐cell‐derived lymphomas [19]. Corticosteroids can penetrate cell membranes and, upon binding to receptors, enter the cell nucleus. This activation process can both induce programmed cell death and suppress the production and release of various inflammatory mediators (such as cytokines). This disrupts the tumor cell's survival environment. It induces apoptosis in B lymphocytes, leading to a marked decrease in the number of cells in the biopsy tissue or even their disappearance. Focal badness and interstitial fibrosis, loss of cellular immunophenotype and altered molecular features are present, leading to misdiagnosis of reactive hyperplasia or missed diagnosis of lymphoma [20]. Coinfections and drug interactions may interfere with diagnosis, especially in the HIV population. The preoperative application of corticosteroids in this case led to necrosis, hemorrhage and partial fibrosis of the pituitary lesion, which interfered with the clinical diagnosis. Intraoperatively, the tumor tissue was seen to be yellowish‐white and hard texture may all be related to this. In histopathology, most of it was necrotic, with only a few CD20+ B cells. Combined with a history of medication, a final diagnosis of lymphoma of B‐cell origin was made. No specific staging was identified because the patient refused further testing such as molecular pathology. Given that a minority of tumor cells express CD34, the possibility of B‐cell lymphoblastic lymphoma must still be considered. According to the WHO 2021 guidelines, immunodeficiency‐associated CNS lymphoma is now a separate classification from CNS primary diffuse large B‐cell lymphoma [21].

Currently, chemotherapy is the mainstay of treatment for lymphoma. Whole‐brain radiotherapy, BTK inhibitors and immunotherapy have demonstrated efficacy in treating CNS lymphoma. Recent studies report encouraging outcomes from the combination of whole‐brain radiotherapy with CAR‐T cell therapy [22]. The prognosis for patients with PCNSL is dismal when compared to patients with systemic lymphoma. A statistical analysis from the United States found a 5‐year survival rate of only 9% for PCNSL associated with HIV/AIDS and 26.2% for PCNSL not associated with HIV/AIDS infection. After widespread use of HAART, the 5‐year survival rate for HIV‐infected cases increased only marginally to 15.8% [23]. There is currently no statistical analysis of the prognosis of pituitary lymphoma in the literature due to its low incidence.

## 5 Conclusion

PCNSL is uncommon, with pituitary lymphoma being even rarer. It may lead to hypopituitarism and cranial nerve dysfunction. Pituitary lymphoma should be considered when imaging findings are inconsistent with conventional pituitary adenoma and involve the pituitary stalk and hypothalamus. When clinical features and imaging studies fail to provide an accurate diagnosis, a multidisciplinary team comprising endocrinology, neurosurgery and radiology should be convened at the earliest opportunity to analyze and discuss the clinical information.

When a definitive diagnosis remains elusive, an organ biopsy should be performed at the earliest opportunity. Prior to biopsy procedures, the administration of corticosteroids may alter imaging signals, further complicating accurate differentiation and increasing the rate of misdiagnosis. Definitive diagnosis requires histopathology, immunohistochemistry, and molecular pathological typing.

## Author Contributions

**Yahui Cui:** conceptualization, project administration, writing – review and editing. **Yuxiao Yuan:** resources. **Liangyan Jin:** writing – review and editing. **Xiantu Zhang:** resources. **Hui Hou:** conceptualization, project administration, resources, writing – original draft.

## Funding

The authors have nothing to report.

## Ethics Statement

This research has been approved by the Ethics Committee of Hangzhou Xixi Hospital, with the approval document number: 2025‐048.

## Consent

Written informed consent was obtained from the patient to publish this report in accordance with the journal's patient consent policy.

## Conflicts of Interest

The authors declare no conflicts of interest.

## Data Availability

Data is provided within the manuscript.

## References

[1] S. Hoffman , J. M. Propp , and B. J. McCarthy , “Temporal Trends in Incidence of Primary Brain Tumors in the United States, 1985–1999,” Neuro‐Oncology 8, no. 1 (2006): 27–37. 16443945 10.1215/S1522851705000323 PMC1871920

[2] G. A. C. van der Sanden , L. J. Schouten , J. A. A. M. van Dijck , J. P. van Andel , G. A. van der Maazen , and J.‐W. W. Coebergh , “Primary Central Nervous System Lymphomas: Incidence and Survival in the Southern and Eastern Netherlands,” Cancer 94, no. 5 (2002): 1548–1556. 11920513 10.1002/cncr.10357

[3] P. U. Freda and K. D. Post , “Differential Diagnosis of Sellar Masses,” Endocrinology and Metabolism Clinics of North America 28, no. 1 (1999): 81–117. 10207686 10.1016/s0889-8529(05)70058-x

[4] J. Gsponer , N. D. Tribolet , J. P. Déruaz , et al., “Diagnosis, Treatment, and Outcome of Pituitary Tumors and Other Abnormal Intrasellar Masses. Retrospective Analysis of 353 Patients,” Medicine (Baltimore) 78, no. 4 (1999): 236–269. 10424206 10.1097/00005792-199907000-00004

[5] M. Krogh‐Jensen , F. D'Amore , M. K. Jensen , et al., “Clinicopathological Features, Survival and Prognostic Factors of Primary Central Nervous System Lymphomas: Trends in Incidence of Primary Central Nervous System Lymphomas and Primary Malignant Brain Tumors in a Well‐Defined Geographical Area. Population‐Based Data From the Danish Lymphoma Registry, LYFO, and the Danish Cancer Registry,” Leukemia & Lymphoma 19, no. 3–4 (1995): 223–233. 8535213 10.3109/10428199509107892

[6] M. Montesinos‐Rongen , A. Brunn , S. Bentink , et al., “Gene Expression Profiling Suggests Primary Central Nervous System Lymphomas to Be Derived From a Late Germinal Center B Cell,” Leukemia 22, no. 2 (2008): 400–405. 17989719 10.1038/sj.leu.2405019 PMC6053313

[7] D. C. Miller , F. H. Hochberg , N. L. Harris , M. L. Gruber , D. N. Louis , and H. Cohen , “Pathology With Clinical Correlations of Primary Central Nervous System Non‐Hodgkin's Lymphoma. The Massachusetts General Hospital Experience 1958–1989,” Cancer 74, no. 4 (1994): 1383–1397. 8055462 10.1002/1097-0142(19940815)74:4<1383::aid-cncr2820740432>3.0.co;2-1

[8] K. E. Therkelsen and A. Omuro , “Advances in Primary Central Nervous System Lymphoma,” Current Neurology and Neuroscience Reports 25, no. 1 (2024): 5. 39585484 10.1007/s11910-024-01389-0

[9] T. N. Kreisl , K. S. Panageas , E. B. Elkin , L. M. Deangelis , and L. E. Abrey , “Treatment Patterns and Prognosis in Patients With Human Immunodeficiency Virus and Primary Central System Lymphoma,” Leukemia & Lymphoma 49, no. 9 (2008): 1710–1716. 18661394 10.1080/10428190802238560

[10] D. Sautner , W. Saeger , and D. K. Ludecke , “Tumors of the Sellar Region Mimicking Pituitary Adenomas,” Experimental and Clinical Endocrinology 101, no. 5 (1993): 283–289. 8299704 10.1055/s-0029-1211245

[11] E. S. Jaffe , S. H. C. E. Swerdlow , E. Campo , et al., WHO Classification of Tumors of the Hematopoietic and Lymphoid Tissues, vol. 2 (IARC Press, 2008).

[12] M. Yasuda , N. Akiyama , S. Miyamoto , et al., “Primary Sellar Lymphoma: Intravascular Large B‐cell Lymphoma Diagnosed as a Double Cancer and Improved With Chemotherapy, and Literature Review of Primary Parasellar Lymphoma,” Pituitary 13, no. 1 (2010): 39–47. 19707877 10.1007/s11102-009-0196-9

[13] W. Kuker , T. Nägele , A. Korfel , et al., “Primary Central Nervous System Lymphomas (PCNSL): MRI Features at Presentation in 100 Patients,” Journal of Neuro‐Oncology 72, no. 2 (2005): 169–177. 15925998 10.1007/s11060-004-3390-7

[14] S. A. Nabavizadeh , A. Vossough , M. Hajmomenian , R. Assadsangabi , and S. Mohan , “Neuroimaging in Central Nervous System Lymphoma,” Hematology/Oncology Clinics of North America 30, no. 4 (2016): 799–821. 27443998 10.1016/j.hoc.2016.03.005

[15] F. Caranci , G. Leone , A. Ponsiglione , et al., “Imaging Findings in Hypophysitis: A Review,” La Radiologia Medica 125, no. 3 (2020): 319–328. 31863360 10.1007/s11547-019-01120-x

[16] A. V. Vorontsov , D. M. Babaeva , V. P. Vladimirova , et al., “Clinical and Radiological Diagnosis of Hypophysitis: A Review of Literature and Own Data,” Problemy Endokrinologii 68, no. 2 (2022): 16–33. 10.14341/probl12777 PMC9764276 35488753

[17] K. Hoang‐Xuan , E. Bessell , J. Bromberg , et al., “Diagnosis and Treatment of Primary CNS Lymphoma in Immunocompetent Patients: Guidelines From the European Association for Neuro‐Oncology,” Lancet Oncology 16, no. 7 (2015): e322–e332. 26149884 10.1016/S1470-2045(15)00076-5

[18] P. V. Naser , M. C. Maurer , M. Fischer , et al., “Deep Learning Aided Preoperative Diagnosis of Primary Central Nervous System Lymphoma,” iScience 27, no. 2 (2024): 109023. 38352223 10.1016/j.isci.2024.109023 PMC10863328

[19] D. Brandsma and J. Bromberg , “Primary CNS Lymphoma in HIV Infection,” Handbook of Clinical Neurology 152 (2018): 177–186. 29604975 10.1016/B978-0-444-63849-6.00014-1

[20] K. Tosefsky , A. D. Rebchuk , K. C. Martin , D. W. Chen , S. Yip , and S. Makarenko , “Preoperative Corticosteroids Reduce Diagnostic Accuracy of Stereotactic Biopsies in Primary Central Nervous System Lymphoma: A Systematic Review and Meta‐Analysis,” Neurosurgery 95, no. 4 (2024): 740–750. 38865324 10.1227/neu.0000000000002944

[21] D. N. Louis , A. Perry , P. Wesseling , et al., “The 2021 WHO Classification of Tumors of the Central Nervous System: A Summary,” Neuro‐Oncology 23, no. 8 (2021): 1231–1251. 34185076 10.1093/neuonc/noab106 PMC8328013

[22] M. S. Shiels , R. M. Pfeiffer , C. Besson , et al., “Trends in Primary Central Nervous System Lymphoma Incidence and Survival in the US,” British Journal of Haematology 174, no. 3 (2016): 417–424. 27018254 10.1111/bjh.14073 PMC4961566

[23] H. Shi , P. Zheng , Z. Fu , et al., “Whole Brain Radiotherapy Combined With CART‐cell Therapy for Relapsed/Refractory Central Nervous System B‐Cell Lymphoma,” Annals of Hematology 104, no. 4 (2025): 2495–2505. 40278918 10.1007/s00277-025-06378-y PMC12052746

